# CHARTING NEW TERRITORIES: EXPLORING THE ROLE OF MICRO-CREDENTIALS IN GERONTOLOGY EDUCATION

**DOI:** 10.1093/geroni/igae098.1000

**Published:** 2024-12-31

**Authors:** M Aaron Guest

**Affiliations:** Chair

## Abstract

In the ever-evolving landscape of higher education, educators constantly aim to meet and anticipate growing demands. This includes catering to learners’ interests while also aligning with the skill sets sought by employers in an ever-evolving economic environment. Flexibility has become paramount, leading to new methods for demonstrating competency and acquiring knowledge and skills. One such approach has been the development of micro-credentials. Micro-credentials signify competency in specific skill sets or areas of knowledge acquired through focused and concise learning experiences. While prevalent in other fields, such as computer science, their emergence in gerontology is relatively recent. Their growth requires us to consider the purpose and objectives of a gerontology-infused curriculum, exploring how they may challenge existing paradigms while fostering new approaches to learning. Guest et al. will provide an overview of gero-micro-credentials and their utilization within private and public sectors. Bronwyn will share a university-based program, emphasizing how these credentials fulfill an emerging need for ongoing education. Heyn highlights the potential impact of these credentials at Hispanic Serving Institutions, particularly regarding opportunities within resource-limited environments. Ellis will then lead our discussion by exploring the undefined nature of these credentials, indicating the necessity for further research and evaluation. With reference to these exemplars, we discuss the strengths and considerations of micro-credentials in the field of aging and work to identify a consensus on how we should engage with and leverage these credentials for advancing gerontology education and practice.

